# Physiology of Angina and Its Alleviation With Nitroglycerin: Insights From Invasive Catheter Laboratory Measurements During Exercise

**DOI:** 10.1161/CIRCULATIONAHA.116.025856

**Published:** 2017-03-28

**Authors:** Kaleab N. Asrress, Rupert Williams, Timothy Lockie, Muhammed Z. Khawaja, Kalpa De Silva, Matthew Lumley, Tiffany Patterson, Satpal Arri, Sana Ihsan, Howard Ellis, Antoine Guilcher, Brian Clapp, Philip J. Chowienczyk, Sven Plein, Divaka Perera, Michael S. Marber, Simon R. Redwood

**Affiliations:** From King’s College London British Heart Foundation Centre of Excellence, Rayne Institute, St. Thomas’ Hospital, London, United Kingdom (K.N.A., R.W., T.L., M.Z.K., K.D.S., M.L., T.P., S.A., H.E., D.P., M.S.M., S.R.R.); National Institute for Health Research Biomedical Research Centre at Guy’s and St Thomas’ NHS Foundation Trust, London, United Kingdom (K.N.A., M.S.M., S.R.R.); Department of Cardiology, Royal North Shore Hospital, Sydney, Australia (K.N.A., K.D.S.); Kolling Institute, Northern Clinical School, University of Sydney, Australia (K.N.A.); Department of Clinical Pharmacology (A.G., P.J.C.) and Division of Imaging Sciences and Biomedical Engineering, Rayne Institute (S.I., S.P.), St Thomas’ Hospital, King’s College London, London, United Kingdom; Guy’s and St Thomas’ Hospital NHS Foundation Trust, London, United Kingdom (B.C.); and Division of Cardiovascular and Neuronal Remodelling, University of Leeds, United Kingdom (S.P.).

**Keywords:** angina pectoris, coronary circulation, exercise, myocardial ischemia, nitroglycerin, physiology

## Abstract

**Background:**

The mechanisms governing exercise-induced angina and its alleviation by the most commonly used antianginal drug, nitroglycerin, are incompletely understood. The purpose of this study was to develop a method by which the effects of antianginal drugs could be evaluated invasively during physiological exercise to gain further understanding of the clinical impact of angina and nitroglycerin.

**Methods:**

Forty patients (mean age, 65.2±7.6 years) with exertional angina and coronary artery disease underwent cardiac catheterization via radial access and performed incremental exercise using a supine cycle ergometer. As they developed limiting angina, sublingual nitroglycerin was administered to half the patients, and all patients continued to exercise for 2 minutes at the same workload. Throughout exercise, distal coronary pressure and flow velocity and central aortic pressure were recorded with sensor wires.

**Results:**

Patients continued to exercise after nitroglycerin administration with less ST-segment depression (*P*=0.003) and therefore myocardial ischemia. Significant reductions in afterload (aortic pressure, *P*=0.030) and myocardial oxygen demand were seen (tension-time index, *P*=0.024; rate-pressure product, *P*=0.046), as well as an increase in myocardial oxygen supply (Buckberg index, *P*=0.017). Exercise reduced peripheral arterial wave reflection (*P*<0.05), which was not further augmented by the administration of nitroglycerin (*P*=0.648). The observed increases in coronary pressure gradient, stenosis resistance, and flow velocity did not reach statistical significance; however, the diastolic velocity–pressure gradient relation was consistent with a significant increase in relative stenosis severity (k coefficient, *P*<0.0001), in keeping with exercise-induced vasoconstriction of stenosed epicardial segments and dilatation of normal segments, with trends toward reversal with nitroglycerin.

**Conclusions:**

The catheterization laboratory protocol provides a model to study myocardial ischemia and the actions of novel and established antianginal drugs. Administration of nitroglycerin causes changes in the systemic and coronary circulation that combine to reduce myocardial oxygen demand and to increase supply, thereby attenuating exercise-induced ischemia. Designing antianginal therapies that exploit these mechanisms may provide new therapeutic strategies.

## Abstract 2

Supplemental Digital Content is available in the text.

**Editorial, see p 35**

Although a common symptom and described as early as 1785,^1^ the exact mechanisms underlying exercise-induced angina are poorly understood. Nitroglycerin, generally referred to as glyceryl trinitrate, was the first medication used for the treatment of angina, described by Murrel in 1879,^2^ and, in its short-acting forms, remains first-line therapy for most patients today. The mechanisms by which nitrates are purported to exert their antianginal effect are numerous, with effects on preload, afterload, and myocardial perfusion being described.^3–7^

Despite this large body of work, the hierarchical relevance of these observations and applicability to patients with coronary artery disease have been limited by the methods used. We developed a novel protocol whereby patients with coronary artery disease could undergo symptom-limited exercise on the catheterization laboratory table. This enabled, for the first time, the immediate effects of nitroglycerin to be assessed with simultaneous invasive central aortic and coronary hemodynamic measurements. This protocol also served to test the feasibility of using such a paradigm in the investigation of novel and established antianginal therapies.

## Methods

### Study Population

Patients with symptoms of exertional angina and documented coronary artery disease were recruited from routine waiting lists for percutaneous coronary intervention at St. Thomas’ Hospital. Sequential patients were screened for suitability and recruited into 2 cohorts in a serial fashion after modification of the initial single-arm protocol in which all subjects received nitroglycerin by enrollment of a second cohort that did not receive nitroglycerin. Exclusion criteria were unstable symptoms; previous myocardial infarction in the study vessel territory; coronary artery bypass surgery; ejection fraction <50%; known left main stem disease; severe multivessel coronary artery disease or chronic total occlusions; severe renal impairment; paced rhythm; left bundle-branch block; or inability to undertake exercise. All vasoactive medications were stopped 48 hours before the procedure. Subjects gave written informed consent in accordance with the protocol approved by the institutional ethics committee (National Research Ethics Service 08/H0802/39). The study was registered with the National Institute for Health Research UK Clinical Research Network portfolio database (Central Portfolio Management System identifier: 7509).

### Catheterization Protocol

The patient was positioned on the catheterization laboratory table, and the distance to the pedals of the bicycle ergometer was adjusted. The right arm was abducted, supported, and strapped onto a specially modified radial artery support so that the arm did not move during exercise. Patients were catheterized via the right radial artery with a 6F sheath. Weight-adjusted heparin was administered (70 U/kg) intra-arterially. The target artery was cannulated with a standard 6F guide catheter. Vasoactive substances such as nitroglycerin, verapamil, or sedatives were not used during arterial access or diagnostic angiography. A dual pressure and velocity sensor 0.014-in intracoronary wire (Combowire XT, Volcano Corp, San Diego, CA) and single pressure sensor 0.014-in Primewire Prestige intra-aortic wire (Volcano Corp) were connected to the Combomap console (Volcano Corp) and advanced to the tip of the guiding catheter. The pressure signals were then normalized. The guide catheter was then inserted into the coronary ostium, and the Combowire was advanced distal to the stenosis of the target coronary artery and manipulated to optimize the Doppler velocity trace. The guide catheter was then disengaged from the ostium, and the pressure wire was passed into the aortic root to record high-fidelity central arterial pressure. All signals were sampled at 200 Hz and stored on a disk for offline analysis. The raw data were extracted and imported into the custom-made CardiacWaves program (King’s College London, London, UK), which used 5-beat averaged signals to calculate all the metrics.

### Exercise Protocol

Once the coronary wires were in place, baseline measurements were taken before the patients underwent exercise on a supine bicycle ergometer. The exercise protocol was a standardized incremental program based on the patient’s weight and age,^8^ typically starting at 25 W and increasing by 20 W each minute with cadence fixed at 60 rpm. Exercise was continued until the development of symptoms of myocardial ischemia or the limits of their performance. Patients then continued to exercise at this workload, at the same cadence and resistance, while nitroglycerin was administered to half the population (2 puffs sublingually at a total dose of 800 μg), and all patients were encouraged to exercise for another 2 minutes. Exercising continuously beyond the ischemic threshold has been shown to be feasible and safe.^9^

### Data Analysis

All patients had continuous 12-lead ECG monitoring throughout exercise, which was analyzed offline by investigators blinded to patient characteristics, exercise time, and hemodynamic conditions. ST-segment depression was measured 80 milliseconds after the J point.

### Pulse Wave Analysis of Central Aortic Pressure

**Figure 1.**
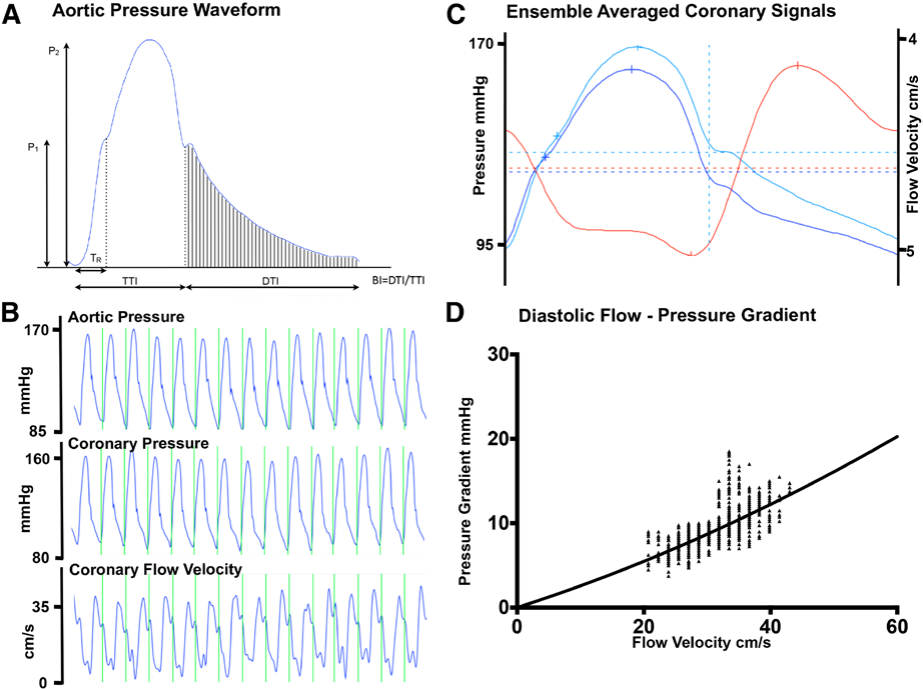
**Aortic and coronary signals.** **A**, Pressure waveform recorded from the ascending aorta from a study patient. Two systolic peaks are labeled P_1_ and P_2_. Augmentation index is calculated as augmented pressure (P_2_−P_1_) as a percentage of total pulse pressure, P_1_. The time to the reflected wave from the foot of the pressure tracing to the inflection point is shown as T_R_. The area under the curve during systole is the tension-time index (TTI), and the area under diastole is the diastolic time index (DTI). Buckberg index is calculated as BI=DTI/TTI. **B**, Typical coronary pressure and flow velocity recordings. **C**, Ensemble-averaged pressure and flow velocity signals (blue lines indicate pressure; red line indicates flow velocity). **D**, The diastolic velocity–pressure gradient relationship, expressed as raw signals, is shown (**bottom left**), with the data fitted with a quadratic equation *Δ*P=0+kv+Sv^2^, where Δ*P* is the instantaneous pressure gradient (mm Hg), v is the coronary flow velocity (cm/s), k is the coefficient of pressure loss resulting from viscous friction, and S is the coefficient of pressure loss caused by flow separation or localized turbulence.

Central arterial pressure waveforms were obtained from the pressure wire positioned in the aortic root and were analyzed with the custom CardiacWaves program (King’s College London). The tension-time index (TTI), diastolic time index (DTI), Buckberg index (BI; calculated as DTI/TTI), and diastolic time fraction were determined, with the dicrotic notch signifying the onset of diastole. Figure 1A shows a typical aortic pressure waveform denoting the measured metrics. TTI relates to myocardial oxygen demand,^10^ and DTI and BI relate to coronary perfusion.^11^ The rate-pressure product was determined as a product of central systolic blood pressure and heart rate, another surrogate of myocardial oxygen consumption.^12^ Augmentation index, a measure of central systolic blood pressure augmentation, and timing of the reflected pressure wave were also determined (Figure 1A). Augmentation index is related to arterial stiffness and likely arises from pressure wave reflection from the aorta, proximal elastic arteries, and peripheral muscular arteries.^13^ Left ventricular (LV) ejection time was measured from the upstroke of the arterial tracing until the trough of the dicrotic notch.

### Coronary Artery Hemodynamics

Mean coronary blood flow velocity (U) was determined from the Doppler signal distal to the coronary stenosis (Figure 1B). Indexes of coronary stenosis severity were calculated to include stenosis resistance as (Pa–Pd)/U, where Pa is aortic pressure and Pd is distal coronary pressure; Pd/Pa ratio; and change in coronary flow velocity (average peak velocity at each exercise time divided by baseline average peak velocity).^14,15^ Microvascular resistance was calculated as Pd/U.^15^

Wave intensity analysis was performed with established methodology.^16–19^ Further description of the technique is found in the Methods section and Figure I of the online-only Data Supplement.

The diastolic flow velocity–pressure gradient relation was calculated to gain further understanding of coronary hemodynamics with the use of established methods.^20–22^ An adjustment was made for the time delay between the digitally archived pressure and velocity signals (55 milliseconds). Representative beats (minimum, 5) at each time point were chosen. For each beat, the flow velocity values from mid diastole to atrial contraction were plotted against the instantaneous pressure gradient (Pa–Pd). The data were then fitted with a quadratic equation, *Δ*P=0+kv+Sv^2^,where *Δ*P is the instantaneous pressure gradient in millimeters of mercury, v is the coronary flow velocity in centimeters per second, k is the coefficient of pressure loss resulting from viscous friction, and S is the coefficient of pressure loss caused by flow separation or localized turbulence (Figure 1D).^20–22^ The values for k and S for each patient’s time point and hemodynamic condition were determined with the least-squares method (constrained to k≥0 and S≥0).

The investigators who performed the data analyses were blinded to all of the clinical patient data.

### Statistical Analysis

Continuous variables were tested for normality with visual inspection (histograms and the normal Q-Q plot) and the Shapiro-Wilk test and expressed as mean±SEM. Repeated-measures ANOVA was used to evaluate the main time trends across the exercise period. If the overall test for the main effect of exercise exertion reached significance in the ANOVA, separate time points were evaluated with paired *t* tests. A correction was not applied for multiple comparisons to reduce the chance of missing significant hemodynamic changes in this exploratory study (type II error). A value of *P*<0.05 was considered statistically significant. Statistical analysis was performed with IBM SPSS version 21.

The study was powered to ensure that there was a sufficient number of patients to detect an attenuation of angina with the administration of nitroglycerin measured by 1 mm of ST-segment shift. On the basis of the results of a similar study from our institution evaluating warm-up angina,^23^ this gave a sample size of 18 patients in each group to achieve 99% power with a probability of a type 1 error of 0.001. This level of power was chosen because it was likely that multiple hemodynamic variables contributed to the effects of nitroglycerin and their variance was possibly greater than that of the ST-segment shift.

## Results

Forty patients (34 male; age, 65.2±7.6 years) completed the protocol, 21 in the nitroglycerin group and 19 in the control group. A total of 56 patients consented to the study but did not complete the protocol. Reasons for noncompletion were as follows: 9 had left main or 3-vessel disease, 4 had chronic total occlusions, 20 had angiographically normal or only minor disease, 3 experienced radial spasm necessitating use of nitroglycerin, 3 were unable to cycle, 3 had very tight lesions where flow was compromised on passing of the wire necessitating immediate percutaneous coronary intervention, and 4 had taken nitrates tablets, 5 had taken β-blockers, and 2 had taken a calcium channel antagonist within 48 hours of the study. Three patients had their research procedures canceled because of a medical emergency in another patient.

**Figure 2.**
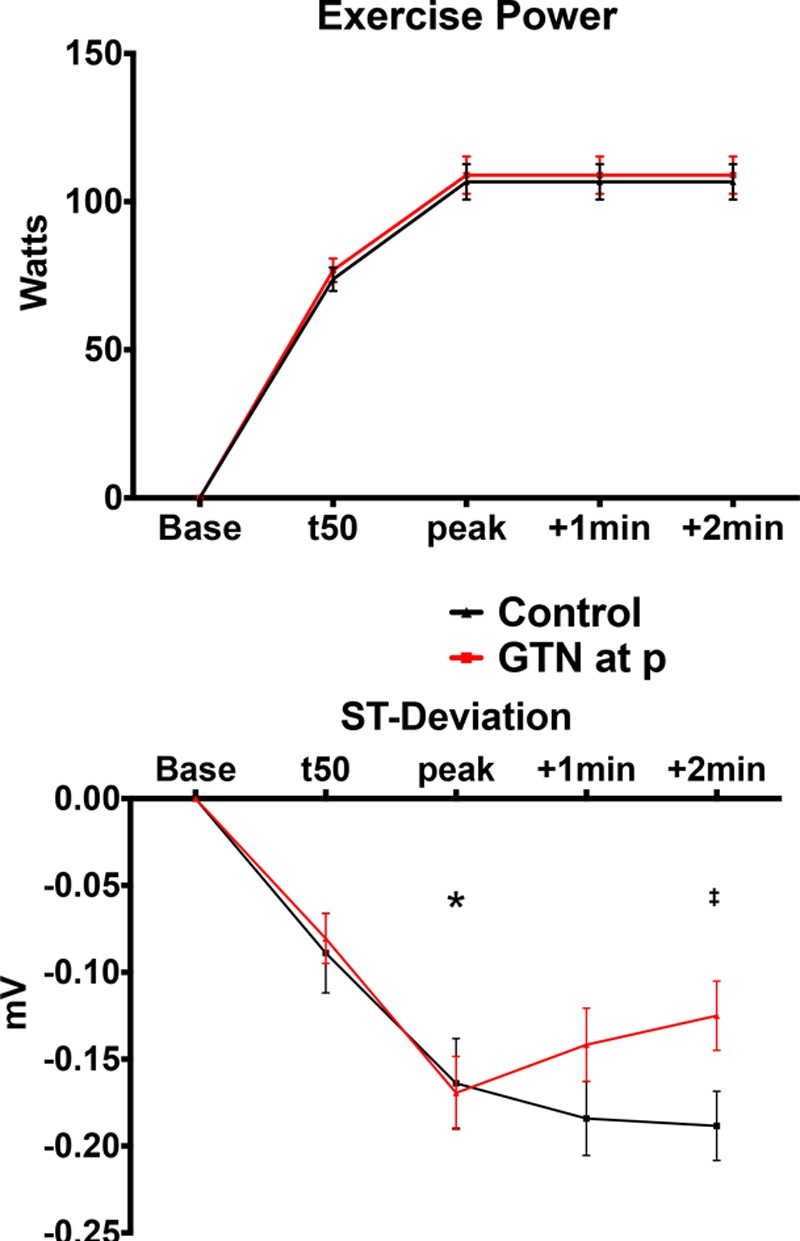
**Supine exercise resistance protocol and ST-segment change.** **Top**, Exercise performance summarized cycling resistance in Watts with patients cycling at 60 rpm, at baseline (Base), 50% of the time (t50) to peak exercise (peak) when patients experienced significant limiting symptoms, and continued exercise 1 and 2 minutes after peak. At time peak, half the patients had sublingual nitroglycerin (GTN) administered, and all patients continued to exercise for an extra 2 minutes at the same workload. **Bottom**, The mean surface ECG ST-segment deviation in millivolts. *Statistical significance, baseline vs peak exercise, *P*<0.05. ‡Significant difference, peak after nitroglycerin (GTN) vs control.

**Figure 3.**
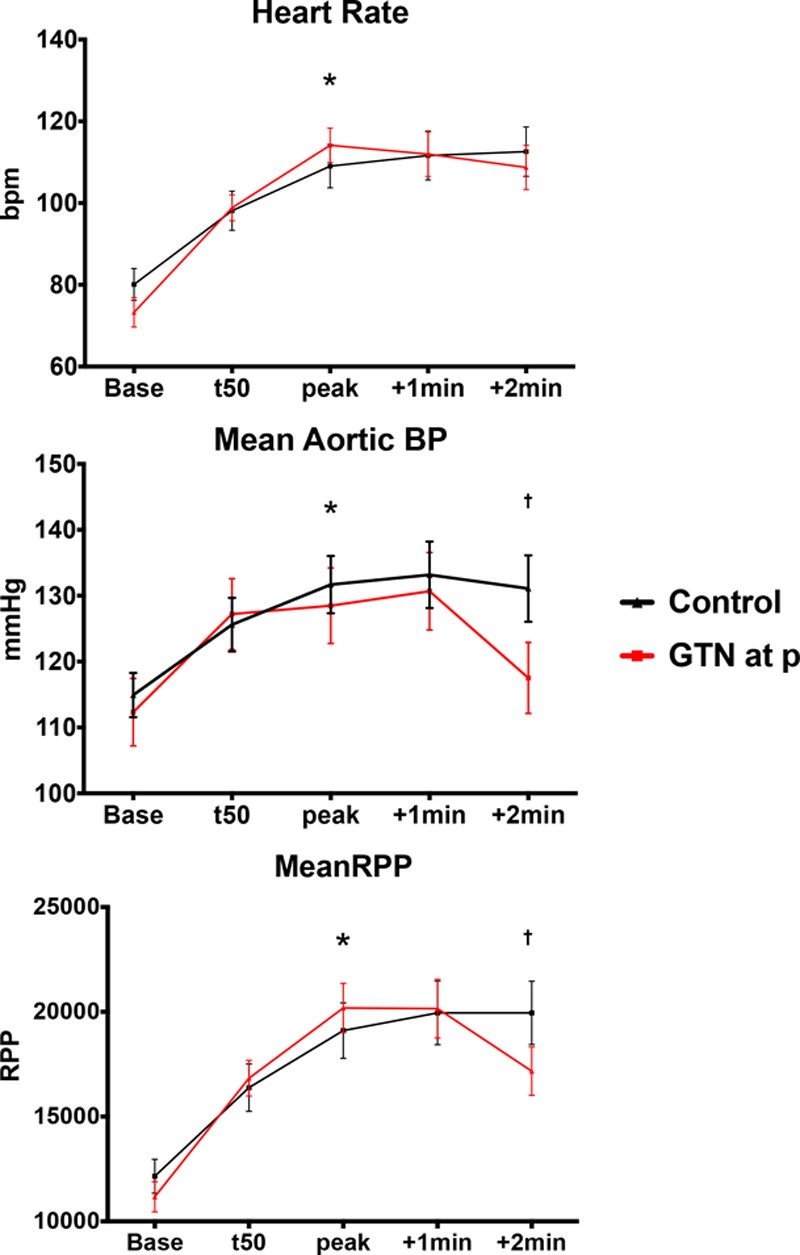
**Hemodynamics on exercise.** Systemic hemodynamic parameters of heart rate, mean aortic pressure, and rate-pressure product (RPP) during study protocol. BP indicates blood pressure; and GTN, nitroglycerin. *Statistical significance, baseline vs peak exercise, *P*<0.05. †Statistically significant difference between peak and 2 minutes after peak.

Baseline demographics of those who completed the study and procedural details are summarized in Tables 1 and 2. Patients in the control and nitroglycerin groups were well matched. Exercise power output and measured ST-segment deviation are summarized in Figure 2. There was a progressive increase in ergometer resistance toward peak exercise, which was maintained at the same workload for a further 2 minutes of exercise in both groups. Limiting symptoms at peak exercise are summarized in Table 3. Despite similar external work in both groups, after nitroglycerin administration, there was a significant attenuation in ST-segment depression. At rest, the ST segments in the territories subtended by the coronary artery lesion were isoelectric, and progressive ST-depression was seen (0.164±0.026 mV for the nitroglycerin group, 0.169±0.021 mV for the control group; *P*=0.836) at peak exercise, consistent with significant ischemia (*P*<0.0001). In the control group, there was numerical worsening of ST-segment depression (*P*=0.355) as patients exercised from the peak period to 2 minutes after nitroglycerin administration, whereas those who received nitroglycerin had improvement in ST-segment depression to 0.119±0.021 mV (*P*=0.003). Figure 3 shows a significant and similar increase in heart rate with exercise between study arms. Systolic blood pressure increased significantly with exercise (*P*<0.0001), with a significant reduction seen 2 minutes after nitroglycerin administration (*P*=0.030), whereas the control group had no significant change (*P*=0.978). Consequently, the rate-pressure product increased significantly with exercise in both groups (*P*<0.0001) but decreased significantly after nitroglycerin (*P*<0.046).

**Table 1.**
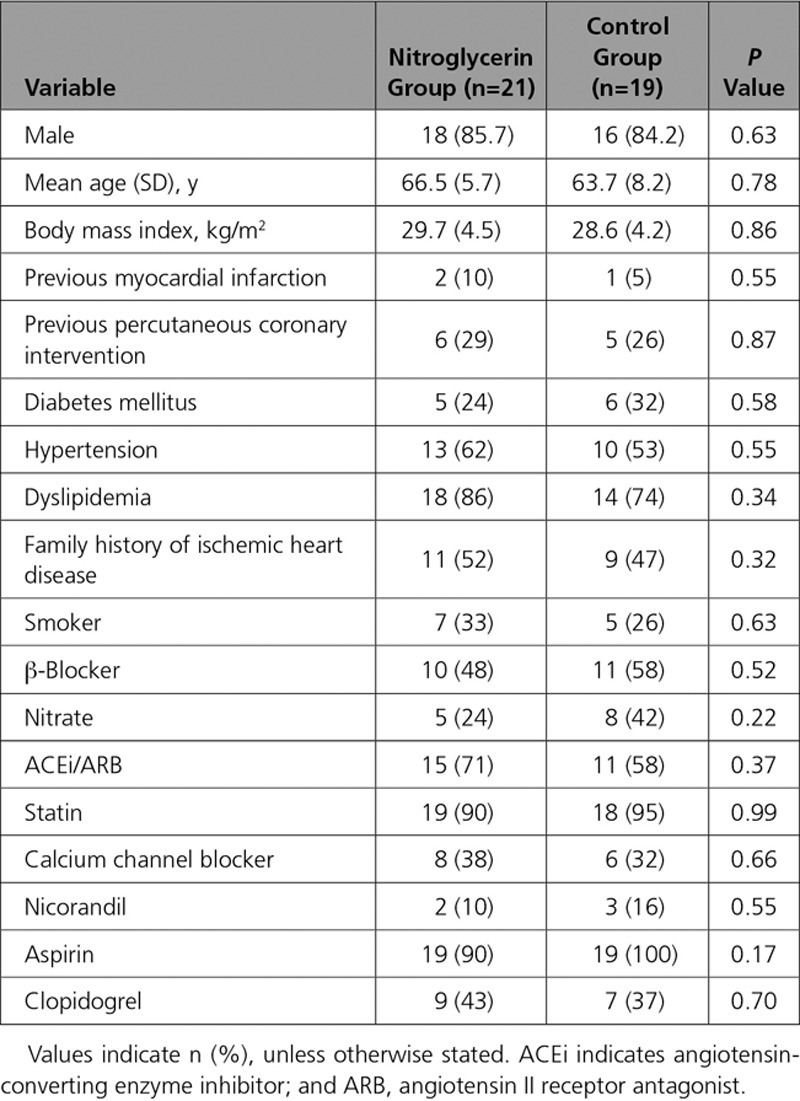
Baseline Demographics

**Table 2.**
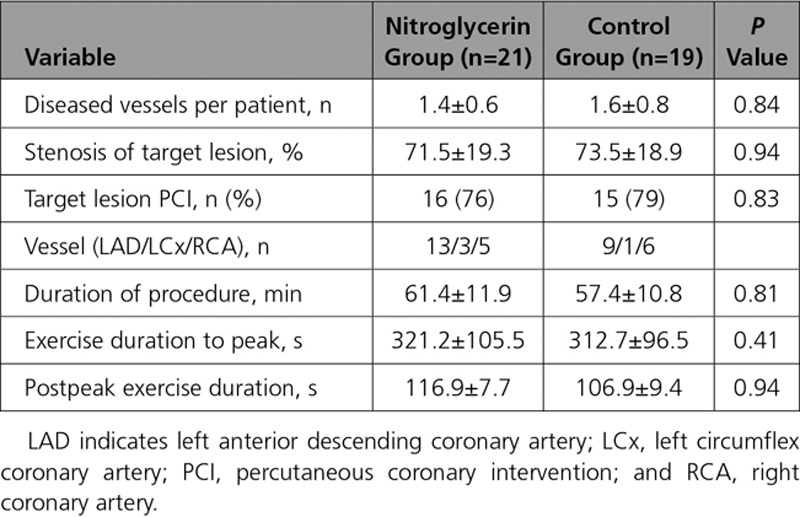
Procedural Details

**Table 3.**
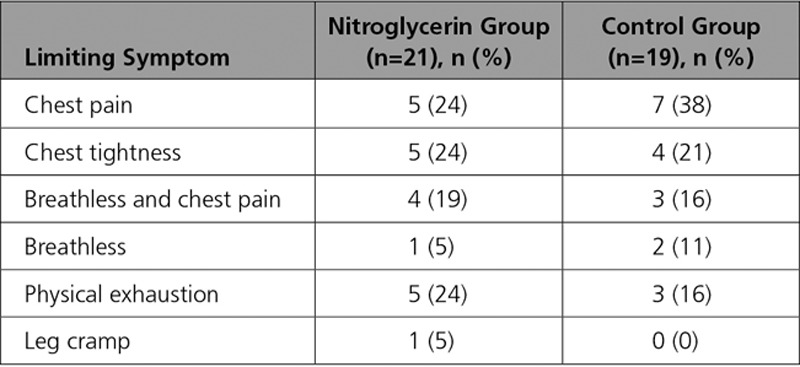
Limiting Symptoms on Exercise

**Figure 4.**
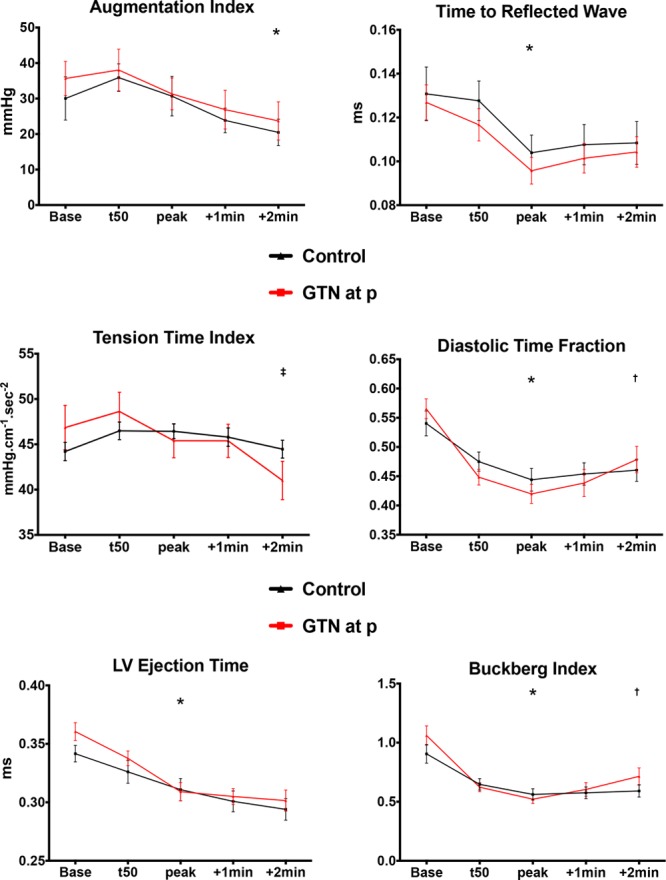
**Indexes of wave reflection and beat cycle physiology.** Systemic hemodynamic augmentation index, time to the reflected wave, tension-time index, diastolic time fraction, left ventricular (LV) ejection time, and Buckberg index. GTN indicates nitroglycerin. *Statistical significance, baseline vs peak exercise, *P*<0.05. †Statistical significance, peak exercise vs 2 minutes after nitroglycerin administration, *P*<0.05. ‡Significant difference, peak exercise after nitroglycerin administration vs control.

Parameters related to wave reflection, DTI, TTI, and BI are summarized in Figure 4. There was no significant change in TTI during incremental exercise (*P*=0.386) because heart rate and blood pressure increased together. However, after nitroglycerin, there was a significant reduction in TTI compared with peak exercise (*P*=0.024) because systolic blood pressure and augmentation index fell. Timing of the reflected pressure wave shortened with exercise (*P*=0.004) but did not significantly change in the peak period, and changes were similar in both groups. There was no significant change in augmentation index to 50% of peak exercise, but significant reductions were seen as exercise continued regardless of treatment group (*P*=0.014 for the nitroglycerin group; *P*=0.033 for the control), contributing to a reduction in afterload. LV ejection time, DTI, BI, and diastolic time fraction all decreased significantly with exercise (*P*<0.05), consistent with increased myocardial oxygen demand and reduced perfusion. After nitroglycerin, there was a significant increase in BI at 2 minutes after administration (*P*=0.0171) and in diastolic time fraction (*P*=0.0260), consistent with improvement in myocardial oxygen supply.

**Figure 5.**
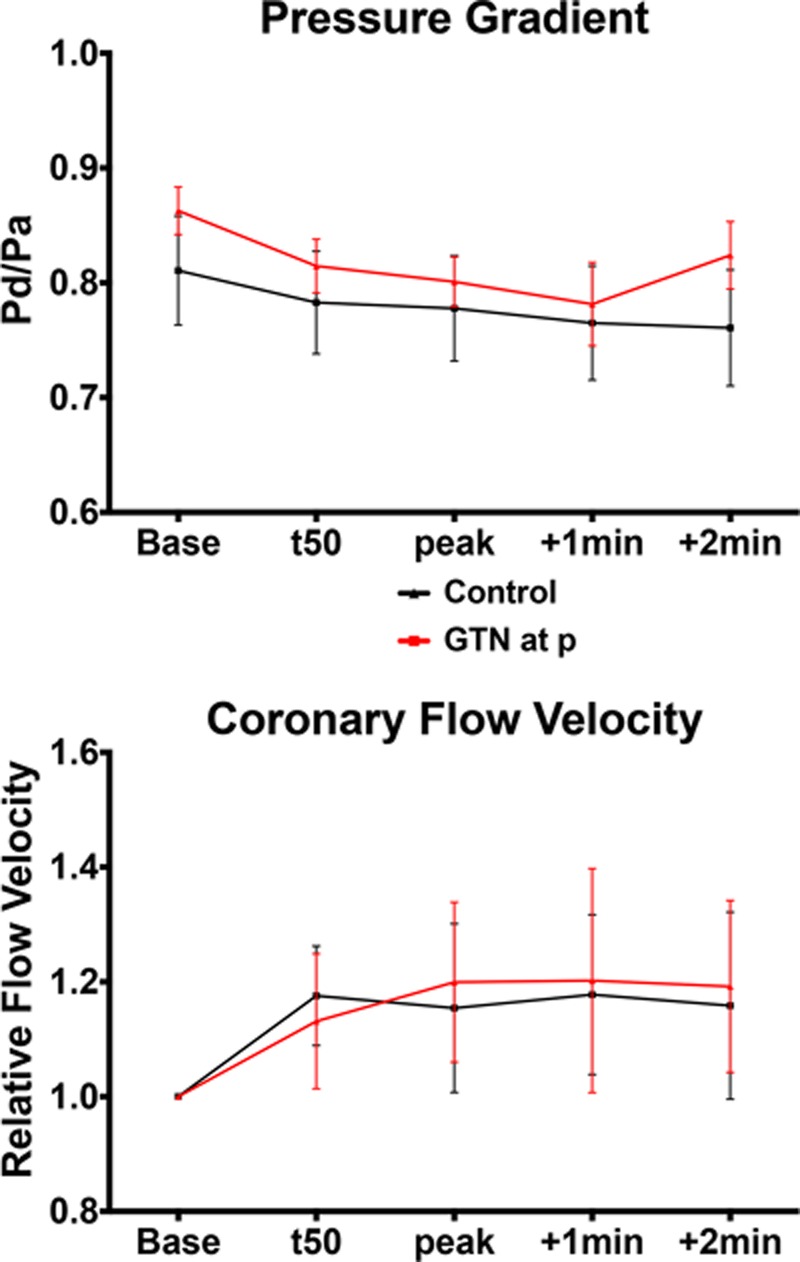
**Coronary hemodynamics.** Mean distal pressure (Pd) to aortic pressure (Pa) ratio and relative change in coronary flow velocity as a ratio of average peak velocity at individual time points vs baseline. GTN indicates nitroglycerin; and t50, 50% of peak exercise.

Intracoronary pressure gradient and flow velocity are shown in Figure 5. There were trends toward worsening of stenosis severity measured by Pd/Pa, as well as an increase in stenosis resistance, but the changes did not reach statistical significance. Coronary flow velocity increased during exercise, but changes were not significant. Similarly, microvascular resistance reduced numerically during exercise, but the difference did not reach statistical significance. Results of the wave intensity analysis are presented in Figure II in the online-only Data Supplement.

**Figure 6.**
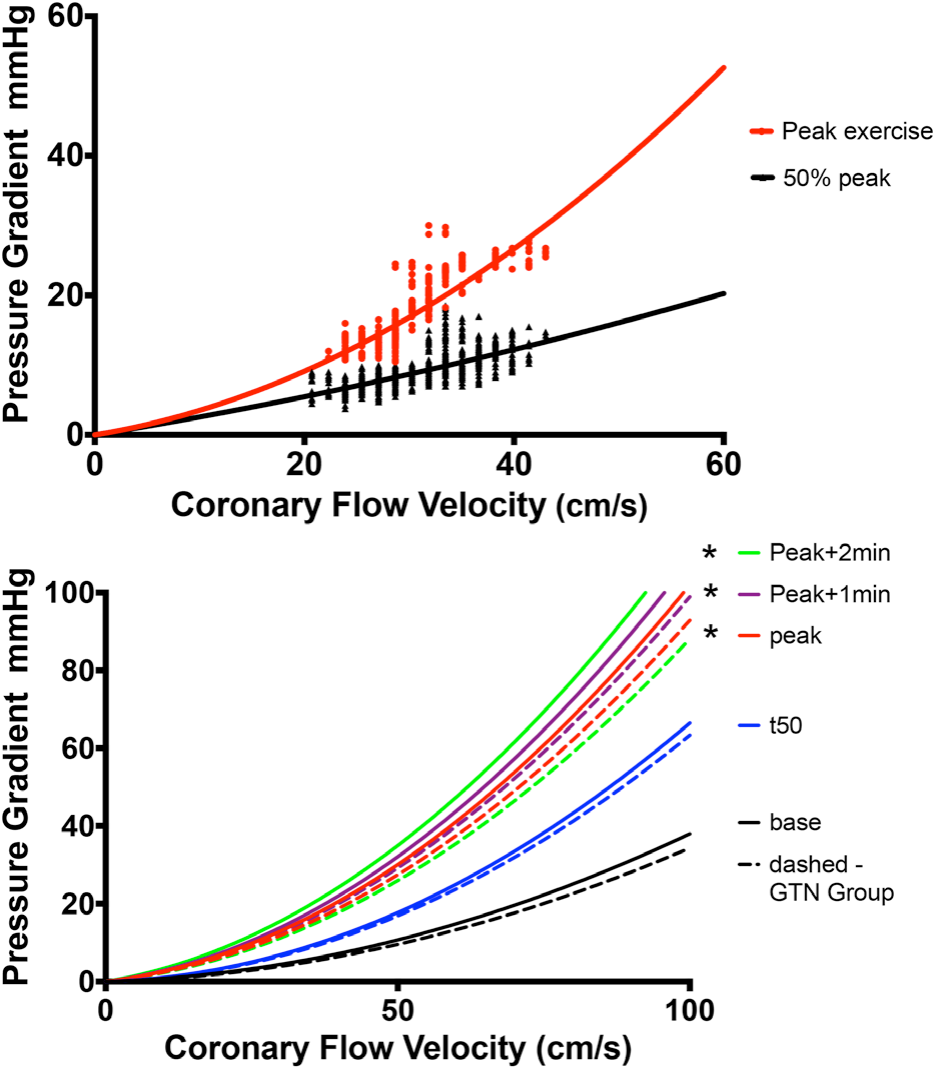
**Diastolic coronary velocity–pressure gradient relation.** **Top**, Two time points: 50% of peak exercise (t50) and peak exercise recordings in 1 individual. **Bottom**, Regression lines that arise from aggregating the k and S coefficients from recordings in all patients. Solid lines represent the control group; dashed lines, those who received nitroglycerin (GTN). Black line indicates baseline; blue, t50; red, peak; purple, 1 minute after peak; and green, 2 minutes after peak. *Statistically significant difference vs baseline of the k coefficient (Table 4 lists all the coefficients).

Figure 6 shows the diastolic velocity–pressure gradient relation. Figure 6A shows a representative example of the raw data plotted for 2 exercise time points in 1 study, at peak and at 50% of peak exercise, for which the regression lines are plotted. Figure 6B shows an aggregate of all the patient studies at each exercise time point between the 2 groups. Table 4 shows a summary of the corresponding k and S coefficients calculated for each time point. The k coefficient was found to be significantly increased between baseline and peak exercise across both groups (*P*<0.0001). However, there were no significant differences between peak exercise and beyond, both within the control group and in those receiving nitroglycerin. The changes seen in the S coefficient did not reach statistical significance, although there are a consistent numeric increase during exercise and a reduction 2 minutes after nitroglycerin administration compared with peak.

**Table 4.**
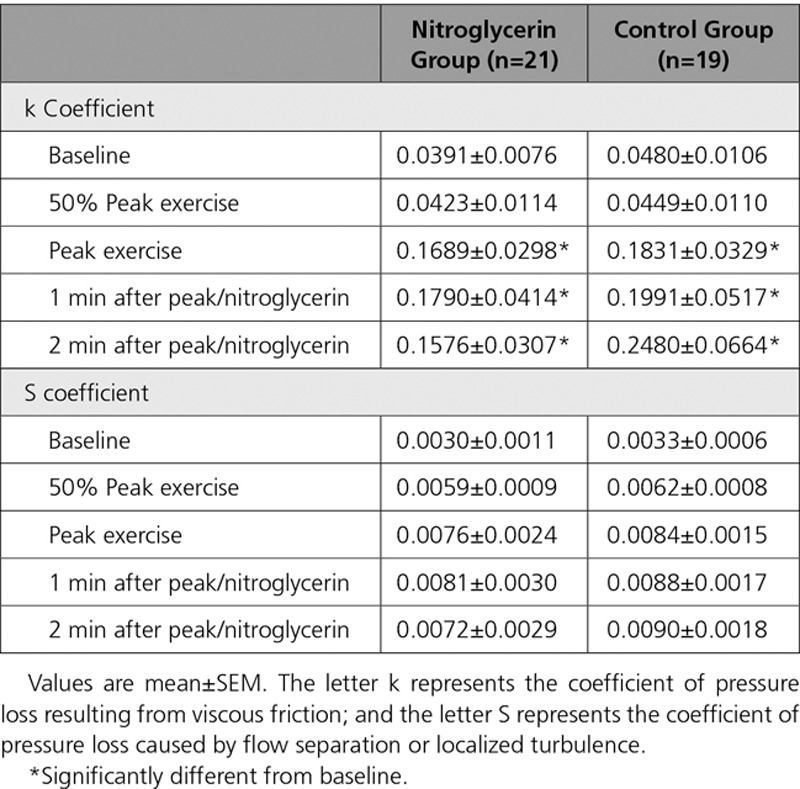
Coefficients Describing the Instantaneous Flow Velocity–Pressure Gradient Relation During Exercise

## Discussion

This study has shown that a catheterization laboratory exercise protocol provides a paradigm with which the physiology of ischemia and the performance of antianginals can be studied. In patients with exercise-induced angina, administration of nitroglycerin causes changes in both the systemic and coronary circulation that combine to alleviate ischemia.

The main findings of the study are that (1) nitroglycerin causes significant reduction in afterload, thereby reducing myocardial oxygen demand; (2) exercise causes reduced peripheral arterial wave reflection, which is not further augmented by the administration of nitroglycerin; (3) exercise causes vasoconstriction of stenosed epicardial segments, increasing relative stenosis severity; (4) nitroglycerin has the effect of vasodilating stenosed segments, thereby reducing relative stenosis severity; and (5) administration of nitroglycerin during exercise-induced ischemia maintains coronary pressure and increases coronary flow, despite a significant reduction in systemic pressure and afterload.

### Myocardial Oxygen Consumption

Our findings suggest that nitroglycerin reduces oxygen consumption by attenuating myocardial work with a reduction in rate-pressure product and TTI. Another major determinant of myocardial oxygen consumption is LV afterload, determined by aortic pressure and the effects of arterial wave reflection. Our results show that there is indeed a significant reduction in arterial pressure augmentation with exercise and further reduction with nitroglycerin, suggesting that both contribute to reductions in afterload by affecting the peripheral arterial circulation.

### Myocardial Perfusion

The hypothesis that nitroglycerin improves oxygen delivery to ischemic tissue by increasing total myocardial blood flow or by producing a redistribution of flow has existed since the 1960s.^24^ The increase in the BI 2 minutes after nitroglycerin administration suggests an improvement in myocardial perfusion. The coronary indexes, including Pd/Pa and stenosis resistance, that have utility in the clinical arena in the assessment of lesion severity do not provide significant insight into the coronary hemodynamics in this study. To fully interpret and understand the dynamics of the coronary circulation, it is important to evaluate the physiology of the epicardial coronary arteries either side of the stenosis, the behavior of the stenosis itself, and the distal microcirculation.

Pioneering work by Gould^20^ and subsequently developed by Marques et al^21^ in describing the diastolic flow velocity–pressure gradient relationship gives some insight into the mechanisms at play. The coefficient k provides an estimate of the pressure loss resulting from viscous friction in the stenotic segment and is dependent on lesion length and relative and absolute percentage stenosis. In this study, the k coefficient is seen to increase significantly during exercise. This suggests that the geometry of the coronary lesion changes and is consistent with a net worsening of lesion severity with exercise. Important work by Gordon and colleagues^25^ who exercised patients with coronary artery disease showed that the anatomic changes on x-ray angiography in response to exercise were dependent on atherosclerotic disease burden whereby stenosed and irregular segments constricted and smooth, disease-free segments dilated. In addition, animal work in smooth arteries suggested dilatation of smooth epicardial segments in response to exercise.^26^ As this occurs, the S coefficient, which is dependent on the relative percentage stenosis and the divergent angle of the stenosis, increases. Although not reaching statistical significance, the trends seen in changes in the S coefficient in this study would support this mechanism. Therefore, the likely unifying coronary mechanism explaining the hemodynamic changes during exercise is that relative stenosis severity increases because of vasoconstriction of the diseased stenotic segments; some vasoconstriction of adjacent irregular, atherosclerotic segments; and vasodilation of the normal segments. Consequently, nitroglycerin has the effect of reducing relative stenosis severity by vasodilating the stenosed and adjacent epicardial segments.

It is important to note that these changes in coronary geometry would have a significant impact on coronary flow, which is not appreciated by the measurement of flow velocity. Our results therefore show that the main effect of nitroglycerin is to maintain distal coronary pressure and to increase coronary flow in the face of reducing aortic pressure and afterload.

The observed worsening of stenosis severity during exercise is likely to be a critical component of exertional symptomatology. This lesion “tone” with exercise may well vary between patients and is unlikely to be picked up by modern catheter-based assessments of lesion severity such as fractional flow reserve^27,28^ in which the administration of adenosine does not change relative stenosis severity. Methods of assessing lesion tone may prove useful in the management of patients with demonstrated coronary artery disease.

A further mechanism responsible for enhanced subendocardial perfusion with nitroglycerin is the transmural redistribution of coronary blood flow resulting from increased diastolic relaxation/delayed systolic compression, as evidenced by the reduced TTI and increased diastolic time fraction and BI after nitroglycerin administration. This is consistent with nuclear studies suggesting that nitrates preferentially redistribute blood to the subendocardium from the subepicardium.^29–31^ This is likely a critical mechanism in reducing ischemia because the autoregulatory mechanisms controlling myocardial blood flow can become exhausted during exercise, rendering the subendocardial layer critically dependent on diastolic time fraction.^20^

### Peripheral Wave Reflection

The effects of exercise in reducing wave reflection in the aortic pressure waveform have been previously described and are due to vasodilatation of the systemic muscular arteries.^5,23,32^ These effects were almost identical to those seen after the administration of nitroglycerin.^5^ Our study demonstrates that there is no additional reduction in arterial wave reflection when nitroglycerin is given during peak exercise, suggesting that it is likely to be governed by the same pathway.

### Limitations

This is a small, single-center study, but it is the first to examine the effects of nitroglycerin by integrating invasive central aortic and coronary hemodynamics during physiological exercise in patients with coronary artery disease. A protocol in which patients continued to exercise at ischemia/near-maximal exertion was used but was feasible to maintain this effort only to ≈2 minutes. The effects of sublingual nitroglycerin are known to persist beyond this time; therefore, our study has the potential to have missed these changes. However, the majority of the hemodynamic effects would be expected to have occurred within the first 2 minutes of administration.^33^

As stated in the Methods, the 2 groups of patients were recruited sequentially and were not randomized; therefore, an element of bias cannot be excluded. The fact that the groups are similar on baseline and procedural factors gives some reassurance, although this remains a limitation.

The aortic and coronary hemodynamics described in this work suggest direct effects of exercise and nitroglycerin on epicardial coronary artery geometry. It was not possible to directly perform coronary angiography during the exercise protocol to allow quantitative assessment of arterial size. In practical terms, the guiding catheter was disengaged from the coronary ostium to prevent catheter-induced trauma during the inevitable movement that occurs in the arm and body during exercise. In addition, injecting contrast medium into coronary arteries gives a short ischemic insult that may have affected the measurements.

We did not measure LV pressure or mechanics and therefore cannot exclude important changes that may have contributed. Furthermore, we used established surrogates of myocardial oxygen consumption and were not able to measure this directly. Although the measurement of LV mechanics and direct measurement of oxygen consumption would provide more definitive mechanistic insight, it was not feasible to take such measurements given the complexity of the protocol already in place.

### Conclusions

In patients with exertional angina and severe coronary artery disease studied during limiting exercise, nitroglycerin produces several changes in systemic, coronary, and LV hemodynamics that combine to reduce ischemia and to enhance cardiac performance. This protocol provides a new paradigm with which the physiology of ischemia and the performance of novel and established antianginals can be studied.

## Sources of Funding

This study was funded by British Heart Foundation Clinical Training Fellowships to Dr Asrress (FS/11/43/28760), Dr Williams (FS/11/90/29087), Dr Lockie (FS/08/058/25305), Dr Khawaja (FS/12/15/29380), and Dr Lumley (FS/13/15/30026); Senior Research Fellowship to Dr Plein (FS/10/62/28409); and Heart Research UK Fellowship to Dr De Silva (RG2593/10/12). All authors acknowledge support from the UK Department of Health via the National Institute for Health Research comprehensive Biomedical Research Center award to Guy’s and St. Thomas’ NHS Foundation Trust.

## Disclosures

None.

## Supplementary Material

## Clinical Perspective

### What Is New?

- The mechanisms governing exercise-induced angina and alleviation by nitroglycerin are incompletely understood.
- The development of a paradigm whereby patients with coronary artery disease can exercise while simultaneously having coronary and central aortic hemodynamics measured invasively has provided a unique opportunity to study these mechanisms.
- Administration of nitroglycerin ameliorates angina by reducing myocardial oxygen demand and increasing supply, with a key component being the reversal of exercise-induced coronary lesion vasoconstriction.

### What Are the Clinical Implications?

- Exercising beyond the onset of ischemia is safe, and the adaptive changes that occur in the peripheral and coronary circulation are of potential benefit, with implications for the design of cardiac rehabilitation and conditioning programs in patients with coronary disease.
- This study highlights the importance of afterload reduction and the use of agents that reduce arterial wave reflection and promote coronary artery vasodilation in the treatment of patients with exercise-induced angina.
- Coronary artery lesion severity is a dynamic process, and varying degrees of lesion tone during exercise is a key component of patient symptomatology; developing strategies to evaluate this clinically may be of benefit.
