# Supplementary material for: Physiology of Angina and Its Alleviation With Nitroglycerin: Insights From Invasive Catheter Laboratory Measurements During Exercise
Source: Circulation. 2017 Mar 28;136(1):24–34. doi: 10.1161/CIRCULATIONAHA.116.025856 (PMC5491223; doi:10.1161/CIRCULATIONAHA.116.025856)
Supplement: Supplementary file 1 [file cir-136-24-s001.pdf]

SUPPLEMENTAL MATERIAL

Physiology of angina and its alleviation with nitroglycerine – insights from invasive catheter laboratory measurements during exercise

Kaleab N Asrress PhD[1,2,3,4]; Rupert Williams PhD[1]; Timothy Lockie PhD[1]; Muhammed Z Khawaja PhD[1]; Kalpa De Silva PhD[1,3]; Matthew Lumley PhD[1]; Tiffany Patterson MB BS[1]; Satpal Arri MB BS[1]; Sana Ihsan MB BS[6]; Howard Ellis MSc[1]; Antoine Guilcher PhD[5]; Brian Clapp PhD[7]; Philip J Chowienczyk MD PhD[5]; Sven Plein MD, PhD[6,8]; Divaka Perera MD[1]; Michael S Marber PhD[1,2]; Simon R Redwood MD[1,2]

Short Title: *Asrress et al*: Nitroglycerine in exercise-induced angina

[1]From the King's College London British Heart Foundation Centre of Excellence, The Rayne Institute, St. Thomas' Hospital, London, UK; [2]National Institute for Health Research Biomedical Research Centre at Guy's and St. Thomas' NHS Foundation Trust, London, UK; [3]Department of Cardiology, Royal North Shore Hospital, Sydney, Australia; [4]The Kolling Institute, Northern Clinical School, University of Sydney, Sydney, Australia; [5]Department of Clinical Pharmacology, King's College London, St Thomas' Hospital, London, UK; [6]Division of Imaging Sciences and Biomedical Engineering, The Rayne Institute, St Thomas' Hospital, King's College London, London, UK; [7]Guy's and St Thomas' Hospital NHS

Foundation Trust, London, UK; [8]The Division of Cardiovascular and Neuronal Remodelling, University of Leeds, Leeds, UK.

Author for correspondence:

Dr Kaleab Asrress MA BM BCh MRCP PhD

Department of Cardiology

5th Floor, Acute Services Building

Royal North Shore Hospital

Reserve Road, St Leonard's, Sydney, NSW, 2065, Australia

E-mail: kaleab.asrress@sydney.edu.au

T: +612 9463 2506

F: +612 9463 2053

Supplement Methods

Wave intensity represents the rate of energy per unit area transported by travelling waves in arteries and is derived from phasic changes in local pressure and flow velocity.[1] In the coronary circulation backward travelling waves are generated distally by cardiac contraction and relaxation at the downstream end, and forward travelling waves arise from changes in aortic pressure at the inlet.[2-4] Therefore, in the coronary circulation Wave Intensity Analysis (WIA) reflects the interactive effects of cardiac mechanics and coronary conductance on coronary haemodynamics.[3,5-7] A typical sequence of waves generated during the cardiac cycle is shown in **Online Figure 1**. The first is a backward compression wave (BCW) an early decelerating pressure wave in early systole during isovolumic contraction. The backward expansion wave (BEW) is an accelerating pressure wave that occurs in early diastolic relaxation at the highest diastolic aortic pressure and max diastolic flow, which is proposed as being the dominant energy in diastole responsible for 'sucking' blood into the microvascular space. The forward waves are related to changes in aortic pressure, the most dominant being the forward compression wave (FCW), a systolic pressure wave that accelerates a very low level mid-systolic flow at peak aortic pressure after isovolumic contraction arising from the increasing proximal pressure as the aortic valve opens.

WIA was performed with custom software (CardiacWaves, King's College London, UK), using methodology previously described.[4,6] The distal pressure and

velocity signals were smoothed with a Savitzky-Golay filter to reduce signal noise [8] and were adjusted to correct for the time delay between the digitally archived pressure and velocity signals (55 ms). The energy carried by the 3 most prominent identified waves, FCW, BCW, BEW, were analyzed and included in this article, and were obtained by the area under the wave and normalized for the sampling interval. The investigators who performed the data analyses were blinded to all of the clinical patient data.

Supplemental Results

The wave intensity during the study protocol is summarized in **Online Figure 2**. Although both the FCW and BCW appear to increase during exercise, the changes are not statistically significant. The BEW increases significantly during exercise (*P*=0.0174 Base to Peak; *P*=0.0018 Base to +2min post GTN). At 2 minutes post peak exercise there was a fall in BEW in the Control group which was not seen in the GTN group (*P*=0.0260).

Supplemental Discussion

The mechanisms of action of GTN have been summarised in the main manuscript. It was felt that the WIA did not provide any additional insight beyond the clear mechanisms already presented. The results are presented here for completeness. Clear changes to coronary geometry have been demonstrated in the main manuscript. WIA has been developed and studied in situations where vessel geometry does not change (certainly intentionally). Although the wave intensity measured at that particular position in the coronary artery is valid, the

wider energetics associated with some of the changes in geometry are less clear. This therefore limits the conclusions that can be reached from this analysis, however the mechanisms identified here, are nevertheless hypothesis generating.

A possible mechanism to aide myocardial perfusion is the effects of microcirculatory suction, which occurs during diastolic relaxation. This is an active processes requiring oxygen. As ischemia has been shown to attenuate diastolic relaxation,[9] we can hypothesise that during ischemia the ability to generate microcirculatory suction is diminished. This is supported by our data where BEW reduced sequentially following peak exercise conditions in the Control group, whereas this is maintained with GTN administration. This is consistent with work by Amende and Fujimoto that showed that nitroglycerine, in the resting state, increased the speed of isovolumic relaxation and improved diastolic function.[10-12]

Supplemental Conclusion

Exercising beyond ischemia causes a reduction in microcirculatory suction likely due to diastolic dysfunction. Administration of GTN results in maintenance of microcirculatory suction which, together with the mechanisms demonstrated in the main manuscript, contributes to the alleviation of angina.

Supplemental Figures

Supplemental Figure 1: Coronary Pressure, Flow and Wave Intensity

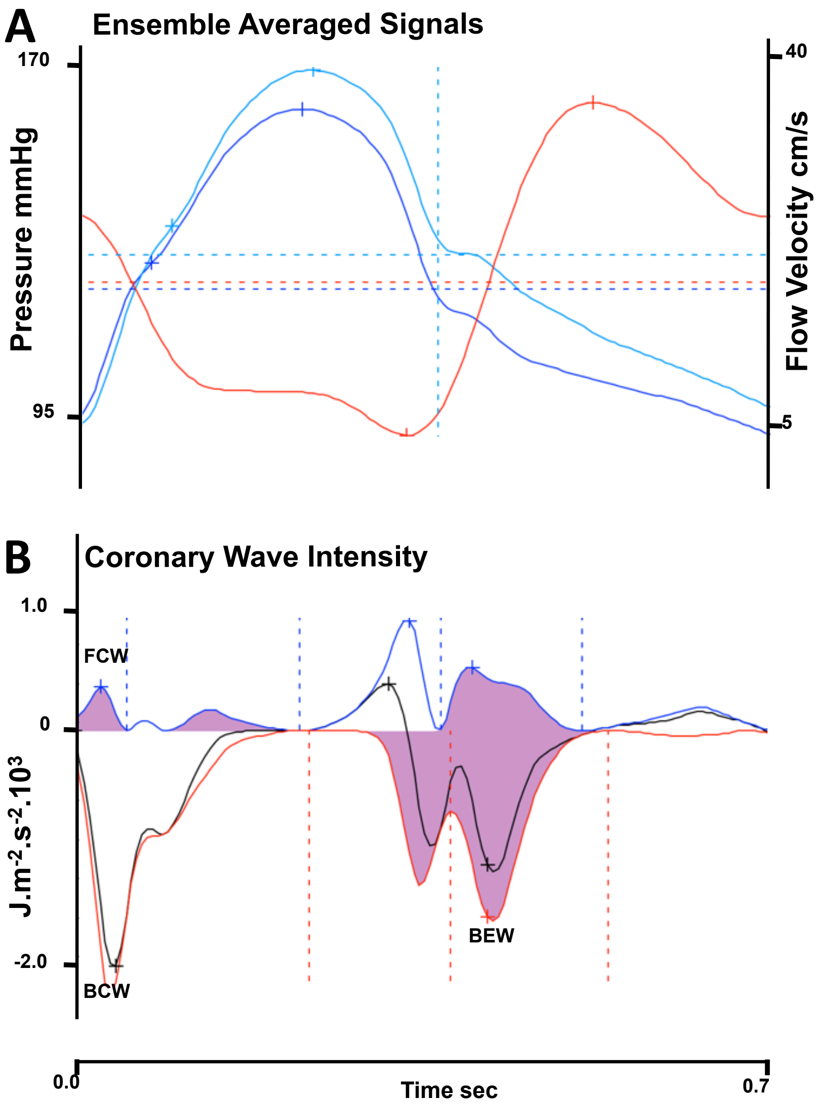

Supplemental Figure 2: Coronary Wave Intensity Analysis

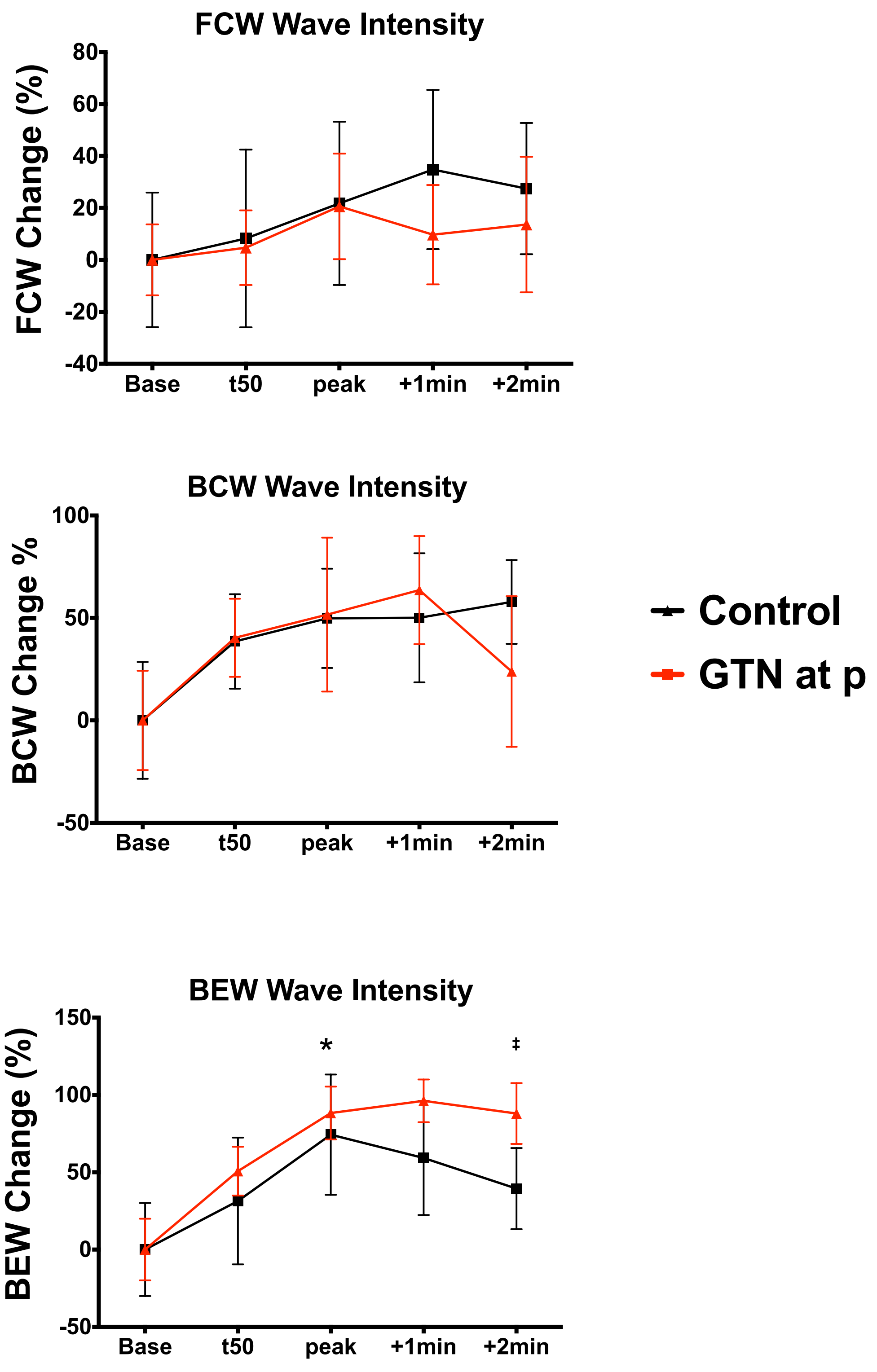

Supplemental Figure Legends

Supplemental Figure 1: Coronary Pressure, Flow and Wave Intensity. A: Coronary pressure (Aortic light blue, distal coronary dark blue line) and flow velocity measurements (red line). B: Calculated wave intensity. The black line outlines the net energies, with the red and blue showing the separated waves. FCW = Forward Compression Wave; BCW – Backward Compression Wave; BEW = Backward Expansion Wave.

Supplemental Figure 2: Coronary Wave Intensity Analysis. FCW = Forward Compression Wave; BCW – Backward Compression Wave; BEW = Backward Expansion Wave. *=statistically significant difference Base vs peak exercise (peak), $P<0.05$, ‡=statistical significant difference at +2min control vs GTN group.

Supplemental References

1. Parker KH, Jones CJ. Forward and backward running waves in the arteries: Analysis using the method of characteristics. *J Biomech Eng* 1990**;112**:322-6.

2**.** Sun YH, Anderson TJ, Parker KH, Tyberg JV. Wave-intensity analysis: A new approach to coronary hemodynamics. *J Appl Physiol* 2000**;89**:1636-44.

3**.** Spaan J, Kolyva C, van den Wijngaard J, ter Wee R, van Horssen P, Piek J, Siebes M. Coronary structure and perfusion in health and disease. *Philos Transact A Math Phys Eng Sci* 2008**;366**:3137-53.

4**.** Davies JE, Whinnett ZI, Francis DP, Manisty CH, Aguado-Sierra J, Willson K, Foale RA, Malik IS, Hughes AD, Parker KH, Mayet J. Evidence of a dominant backward-propagating "suction" wave responsible for diastolic coronary filling in humans, attenuated in left ventricular hypertrophy. *Circulation* 2006**;113**:1768-78.
5**.** Lockie TP, Rolandi MC, Guilcher A, Perera D, De Silva K, Williams R, Asrress KN, Patel K, Plein S, Chowienczyk P, Siebes M, Redwood SR, Marber MS. Synergistic adaptations to exercise in the systemic and coronary circulations that underlie the warm-up angina phenomenon. *Circulation* 2012**;126**:2565-74.
6**.** De Silva K, Foster P, Guilcher A, Bandara A, Jogiya R, Lockie T, Chowiencyzk P, Nagel E, Marber M, Redwood S, Plein S, Perera D. Coronary wave energy: A novel predictor of functional recovery after myocardial infarction. *Circ Cardiovasc Interv* 2013**;6**:166-75.
7**.** Lumley M, Williams R, Asrress KN, Arri S, Briceno N, Ellis H, Rajani R, Siebes M, Piek JJ, Clapp B, Redwood SR, Marber MS, Chambers JB, Perera D. Coronary physiology during exercise and vasodilation in the healthy heart and in severe aortic stenosis. *J Am Coll Cardiol* 2016**;68**:688-97.
8**.** Savitzky A, Golay MJ. Smoothing and differentiation of data by simplified least squares procedures. *Anal Chem* 1964**;36**:1627-39.
9**.** Miyazaki S, Guth BD, Miura T, Indolfi C, Schulz R, Ross J. Changes of left ventricular diastolic function in exercising dogs without and with ischemia. *Circulation* 1990**;81**:1058-70.
1**0.** Amende I, Simon R, Hood WP, Lichtlen PR. Effects of nitroglycerin on left ventricular diastolic properties in man. *Z Kardiol* 1983**;72 Suppl 3**:62-5.

1**1.** Fujimoto N, Onishi K, Tanabe M, Dohi K, Funabiki K, Kurita T, Yamanaka T, Nakajima K, Ito M, Nobori T, Nakano T. Nitroglycerin improves left ventricular relaxation by changing systolic loading sequence in patients with excessive arterial load. *J Cardiovasc Pharmacol* 2005**;45**:211-6.

1**2.** Fujimoto N, Onishi K, Dohi K, Tanabe M, Kurita T, Takamura T, Yamada N, Nobori T, Ito M. Hemodynamic characteristics of patients with diastolic heart failure and hypertension. *Hypertension Research* 2008**;31**:1727.
